# New species and records of *Lobrathium* Mulsant & Rey (Coleoptera, Staphylinidae, Paederinae) from China

**DOI:** 10.3897/zookeys.304.5406

**Published:** 2013-05-27

**Authors:** Wen-Rong Li, Mei-Jun Zhao, Cong-Chao Dai, Li-Zhen Li

**Affiliations:** 1 Department of Biology, College of Life and Environmental Sciences, Shanghai Normal University, Shanghai, 200234, P. R. China

**Keywords:** Coleoptera, Staphylinidae, Paederinae, *Lobrathium*, China, taxonomy, new species

## Abstract

Seven new species of the genus *Lobrathium* Mulsant & Rey from China are described and illustrated: *Lobrathium anatinum* Li & Li, **sp. n.** (Guangxi), *Lobrathium diaoluoense* Li & Li, **sp. n.** (Hainan), *Lobrathium dufui* Li & Li, **sp. n.** (Hubei), *Lobrathium lirunyui* Li & Li, **sp. n.** (Guizhou), *Lobrathium pengi* Li & Li, **sp. n.** (Guangxi), *Lobrathium quyuani* Li & Li, **sp. n.** (Hubei) and *Lobrathium uncinatum* Li & Li, **sp. n.** (Qinghai). A recent key to the species of mainland China is modified to accommodate the new species. New locality data are provided for eleven species.

## Introduction

According to a recent revision (Assing 2012), the genus *Lobrathium* Mulsant & Rey, 1878 is represented in China by 43 species (24 species from mainland China and 20 from Taiwan, with *Lobrathium hongkongense* distributed both in mainland China and Taiwan). Later, Li et al. (2013) described four additional species from mainland China: *Lobrathium quadrum* (Li, Solodovnikov & Zhou 2013), *Lobrathium rutilum* (Li, Solodovnikov & Zhou 2013), *Lobrathium tortuosum* (Li, Solodovnikov & Zhou 2013) and *Lobrathium zonalis* (Li, Solodovnikov & Zhou 2013). Herein, we report seven new species of *Lobrathium* from mainland China and additional locality data for eleven species.

## Material and methods

All the material treated in this study is deposited in the Insect Collection of Shanghai Normal University, Shanghai, China (**SNUC**).

Type labels are cited in their original spelling. A slash (/) is used to separate different labels. Type material bears the following type label: ‘HOLOTYPE [red] or PARATYPE [yellow], [genus name, species name], sp. n., [authors of the species], det. 2013.

The specimens were killed with ethyl acetate and then dried. Materials were stored in 75% ethanol; genitalia and small parts were embedded in Euparal on plastic slides that were attached to the same pin with the specimens.

Morphological studies were carried out using an Olympus SZX 16 stereoscope. A digital camera Canon EOS 50D with MP-E 65 mm Macro Photo Lens was used for the habitus photos. An Olympus CX21 microscope and a digital camera Canon G9 were used for the photos of small structures. The map was created using MapGis.

The measurements of various body parts are abbreviated as follows: **BL**–length of the body from the labral anterior margin to the anal end; **HL**–length of the head from the anterior margin of the frons to the posterior margin of the head; **HW**–maximum width of the head; **PL**–length of the pronotum along the midline; **PW**–maximum width of the pronotum; **EL**–length of the elytra from the anterior margin to the elytral posterior margin along suture; **EW**–maximum width of the elytra; **AL**–length of the aedeagus from the apex of the ventral process to the base of the aedeagal capsule.

## Taxonomy

### Key to the *Lobrathium* species of mainland China (modified from Assing 2012: 84–86)

**Table:** 

1	Elytra with posterior portion partly or completely yellowish or reddish, mostly with yellowish spots, and often with bluish to purple hue	2
–	Elytra uniformly dark-brown to blackish	15a
2	Elytra with more or less extensive yellowish coloration posteriorly, at least posterior two fifths completely yellowish	2a
–	Elytra less extensively yellowish or reddish posteriorly, usually with more or less defined spots often leaving the lateral and/or posterior margins blackish	4
2a	♂: sternite VI with modified, stout and short black setae (Fig. 18D). Qinghai	*Lobrathium uncinatum* Li & Li sp. n.
–	♂: sternite VI without modified, stout and short black setae	2b
2b	♂: sternite VII with modified, stout and short black setae (Fig. 8D). Hubei	*Lobrathium dufui* Li & Li sp. n.
–	♂: sternite VII without modified, stout and short black setae	3
3	♂: posterior excision of sternite VIII smaller and less deep; aedeagus 1.1 mm long, with ventral process apically undivided. Shaanxi: Qinling Shan	*Lobrathium schuelkei* Assing, 2012
–	♂: posterior excision of sternite VIII slightly larger and somewhat deeper; aedeagus larger, 1.2–1.3 mm long, ventral process apically with two long processes. Hubei, Beijing, Shanxi	*Lobrathium taureum* Assing, 2012
4	Large species; length of forebody at least 4.9 mm. Pronotum broad, 1.10–1.20 times as long as broad. Elytra 0.90–0.95 times as long as pronotum and without bluish hue. ♂: sternite VII with distinctly modified, short and stout black setae; sternite VIII with conspicuously deep posterior excision, its depth at least approximately half the length of sternite; ventral process of aedeagus ventrally with rasp-like structures	5
–	Smaller species; length of fore body 4.6 mm at most. Pronotum more slender, 1.20–1.35 times as long as broad, only in one species broader (*Lobrathium radens*). Elytra often longer than pronotum and often with bluish or purple hue. ♂: sternite VII in most species without strongly modified setae; sternite VIII with less deep posterior excision, except in *Lobrathium bispinosum* and *Lobrathium tuberosum*	5a
5	Pronotum slightly broader, 1.10–1.15 times as long as broad. ♂: posterior excision of sternite VIII extremely deep, reaching well beyond middle of sternite; aedeagus 1.6 mm long, ventral process with two rasp-like projections and stoutly blade-shaped. Northern Yunnan: Diancang Shan	*Lobrathium excisissimum* Assing, 2012
–	Pronotum slightly less broad, 1.15–1.20 times as long as broad. ♂: posterior excision of sternite VIII less deep, approximately reaching middle of sternite; aedeagus 1.5 mm long, ventral process with more numerous rasp like projections, somewhat more slender and apically more acute in ventral view. Eastern Guizhou: Leigong Shan	*Lobrathium radens* Assing, 2012
5a	♂: aedeagus 1.70–1.72 mm long, ventral process apically not bifid (Fig. 2C). Guangxi	*Lobrathium anatinum* Li & Li sp. n.
–	♂: aedeagus 1.35 mm long, ventral process apically bifid	6
6	Elytral spots situated in anterior portion of posterior half of elytra (i.e., at some distance from posterior margin). Dorsal surface of head uneven, with median and lateral impressions. Punctation of head and pronotum extremely dense. Relatively large species; length of fore body 4.4–4.6 mm. Antennae slender. ♂: sternite VII moderately transverse; aedeagus 1.35 mm long, ventral process apically bifid. Western Hubei: Daba Shan, Guizhou, Shaanxi	*Lobrathium ablectum* Assing, 2012
–	Elytral spots situated at or near posterior margin of elytra. Dorsal surface of head without distinct impressions. Punctation of head and pronotum less dense. Smaller species, length of fore body usually 4.0 mm at most, except for *Lobrathium spathulatum* (3.7–4.5 mm). Male sexual characters different. A reliable identification of the following species is possible only based on the male sexual characters	6a
6a	♂: bifurcation of the apex of the ventral process of the aedeagus forming an angle of more than 30 degrees in lateral view (Fig. 12B). Guangxi, Shiwanda Shan	*Lobrathium pengi* Li & Li sp. n.
–	♂: bifurcation of the apex of the ventral process of the aedeagus forming an angle of less than 30 degrees in lateral view (Fig. 7B). Hainan	*Lobrathium diaoluoense* Li & Li sp. n.
7	Elytra black, without bluish or purple hue, 0.9–1.0 times as long as pronotum	8
–	Elytra usually with, rarely without bluish or purple hue, 1.0–1.15 times as long as pronotum	12
8	♂: sternite VIII with small posterior excision in asymmetric position, posterior margin with tooth-like projection on either side of excision; aedeagus approximately 1.5 mm long, ventral process very long, slender, and apically asymmetric. Widespread: Sichuan, Shaanxi, Hubei, Guizhou	*Lobrathium tortile* Zheng, 1988
–	♂: sternite VIII with deep posterior excision in symmetric position; aedeagus of different morphology	9
9	♂: aedeagus 0.9–1.0 mm long, ventral process long, slender, apically acute, and very thin at base; sternite VIII with very broad and deep posterior excision, on either side of excision with dense pubescence. Central Sichuan: Qingcheng Shan	*Lobrathium gladiatum* Zheng, 1988
–	Male sexual characters different	10
10	♂: sternite VII anteriorly with tubercle; sternite VIII oblong and with U-shaped posterior excision; aedeagus 1.2 mm long and with massive ventral process. Jiangxi	*Lobrathium tuberosum* Assing, 2012
–	♂: sternite VII without tubercle; posterior excision of sternite VIII of different shape; aedeagus longer, at least approximately 1.4 mm long	11
11	♂: sternite VII with shallow posterior impression with pubescence; sternite VIII with moderately deep posterior excision and of characteristic chaetotaxy; aedeagus 1.4 mm long and with ventral process of distinctive shape. Northeastern Hubei, Zhejiang	*Lobrathium demptum* Assing, 2012
–	♂: sternite VII with more pronounced posterior impression without pubescence; posterior excision of sternite VIII much deeper, broader, and of subtrapezoid shape; aedeagus longer, 1.5 mm long, ventral process with two tooth-like projections ventrally. Southeastern Guizhou, Jiangxi	*Lobrathium bispinosum* Assing, 2012
12	Elytra with weak purple hue; posterior spots relatively small, defined, and of circular shape. Pronotum less oblong, approximately 1.2 times as long as broad. ♂: sternite VII moderately transverse and with moderately deep posterior excision; aedeagus 1.0 mm long, ventral process with dorsal carina and apically acute. Northern Yunnan	*Lobrathium retrocarinatum* Assing, 2012
–	Elytra usually with bluish hue; posterior spots usually larger and/or of different shape or less defined. Pronotum more oblong, at least approximately 1.25 times as long as broad. ♂: sternite VII either with strongly modified short and black setae or without modified setae at all; sternite VIII and aedeagus of different shape	13
13	♂: sternite VII with distinctly modified short and stout black setae; sternite VIII with deep U-shaped posterior excision; aedeagus approximately 1.2 mm long, ventral process of distinctive morphology. Sichuan, Shaanxi, Yunnan	*Lobrathium hebeatum* Zheng, 1988
–	♂: sternite VII without distinctly modified setae; sternite VIII with less deep and differently shaped posterior excision; aedeagus of different morphology	14
14	♂: posterior excision of sternite VII small; aedeagus approximately 1.0 mm long. Widespread and common species: China, Taiwan, southern Japan	*Lobrathium hongkongense* Bernhauer, 1931
–	♂: posterior excision of sternite VII larger and of broadly triangular shape; aedeagus of different shape. Species with more restricted distributions	14a
14a	♂: aedeagus ventral process broader, evenly narrowed and acute apically in ventral view (Fig. 13C). Hubei	*Lobrathium quyuani* Li & Li sp. n.
–	♂: aedeagus ventral process of different shape in ventral view	15
15	♂: aedeagus of somewhat variable shape and size, 1.3–1.5 mm long, ventral process very slender, apically acute, and weakly asymmetric; sternites VII and VIII as in Figs 4D, E. Widespread in China: Shaanxi, Sichuan, Hubei, Yunnan, Qinghai	*Lobrathium configens* Assing, 2012
–	♂: aedeagus longer, 1.6–1.8 mm long, ventral process distinctly asymmetric and apically distinctly dilated (ventral view); sternites VII and VIII as in Figs 14D, E. Widespread in China: Sichuan, Shaanxi, Shanxi, Hubei, Zhejiang	*Lobrathium spathulatum* Assing, 2012
15a	Body reddish to reddish-brown; head posterior angles not marked, punctation fine and dense; eyes very small, one third as long as distance from posterior margin of eyes to neck; elytra 1.25 times as wide as pronotum. ♂: aedeagus 1.56 mm long, ventral process of distinctive morphology (Figs 11A–E). Guizhou	*Lobrathium lirunyui* Li & Li sp. n.
–	Male characters different	16

#### Lobrathium ablectum

Assing

http://species-id.net/wiki/Lobrathium_ablectum

Figs 1

Lobrathium ablectum Assing, 2012: 106. Type locality: creek valley 8 km NW Muyuping, Daba Shan, Hubei.

##### Material examined

(6 ♂♂, 1 ♀)**. China, Shaanxi:** 3 ♂♂, 1 ♀, Hanzhong City, Nanzheng County, Yuanba Town, Liping National F. P., 32°50'N, 106°36'E, 1400–1600 m, 16–VII–2012, Li et al. leg. **Hubei:** 1 ♂,Wufeng County, Houhe National Reserve, 1100 m, 30°04'N, 110°37'E, 30–IV–2004, Li leg. **Guizhou:** 1 ♂, Tongren City, Fanjing Shan, Hu & Tang leg.; 1 ♂, Yanhe County, Wanjia Village, Mayanghe N. R., 900 m, 28°51'N, 108°21'E, 03–X–2007, Zhu leg.

##### Distribution.

The known distribution is confined to the Daba Shan (Assing 2012) and the Wuling Shan in Hubei, Guizhou, and Shaanxi.

**Figure 1.**
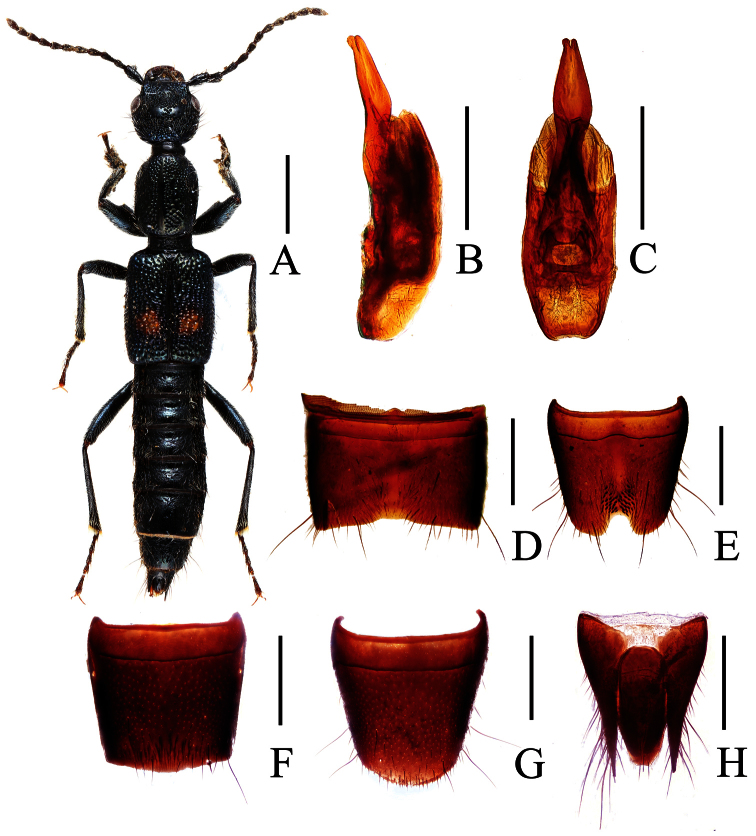
*Lobrathium ablectum*. **A** habitus **B** aedeagus in lateral view **C** aedeagus in ventral view **D** male sternite VII **E** male sternite VIII **F** female tergite VIII **G** female sternite VIII **H** female tergites IX-X. Scales: **A** 1mm, **B**–**H** 0.5mm.

#### Lobrathium anatinum

Li & Li sp. n.

urn:lsid:zoobank.org:act:9D9DA5B9-5138-437B-B2B9-81E536FD2E6E

http://species-id.net/wiki/Lobrathium_anatinum

Figs 2

##### Type material

(2 ♂♂)**. Holotype**, ♂: “China, Guangxi, Lingui County, Anjiangping, 1700 m, 25°33'N, 109°55'E, 17–VII–2011, Peng Zhong leg. / Holotype ♂, *Lobrathium anatinum*, sp. n. Li & Li, det. 2013”. **Paratype**, ♂: “China, Guangxi, Lingui County, Anjiangping, 1400–1700 m, 25°33'N, 109°56'E, 14–VII–2011, Peng Zhong leg. / Paratype ♂, *Lobrathium anatinum*, sp. n. Li & Li, det. 2013”.

##### Description.

**Figure 2.**
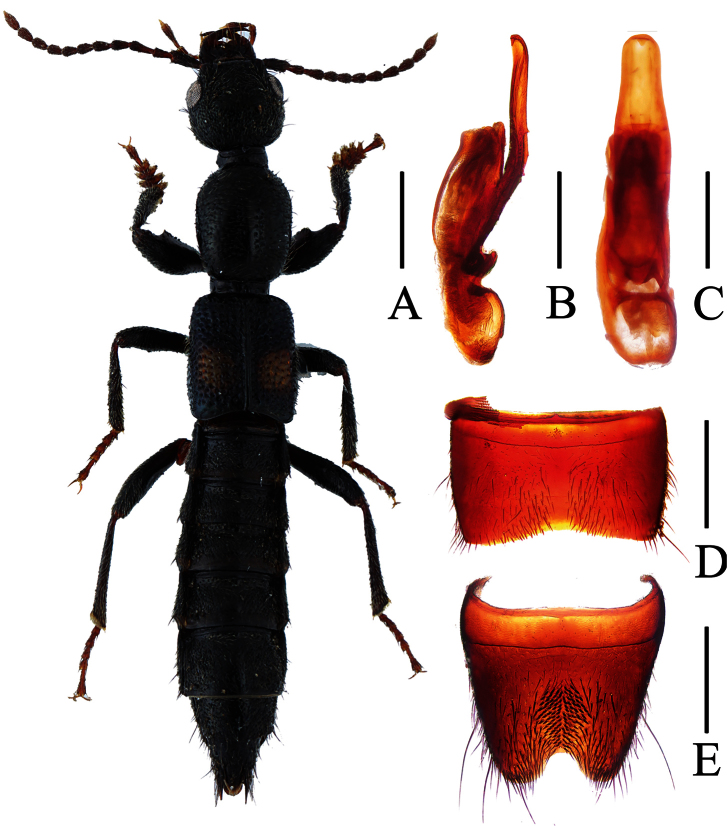
*Lobrathium anatitum*. **A** habitus **B** aedeagus in lateral view **C** aedeagus in ventral view **D** male sternite VII **E** male sternite VIII. Scales: **A** 1mm, **B**–**E** 0.5mm.

Body length 7.28–7.89 mm, length of fore body 3.89–4.0 mm. Habitus as in Fig. 2A. Coloration: body black with distinct bluish hue, middle of elytra with yellowish spot not reaching lateral and posterior margins; legs blackish with paler tarsi, antennae dark brownish to blackish.

Head weakly transverse (HW/HL = 1.10–1.11), widest across eyes; posterior angles broadly rounded; punctation dense and moderately coarse, sparser in median dorsal portion; interstices without microsculpture. Eyes large, more than half as long as distance from posterior margin of eye to neck in dorsal view. Antenna long and slender, 1.96–2.22 mm long.

Pronotum 1.24–1.30 times as long as broad, as wide as head (PW/HW = 1.0), lateral margins convex in dorsal view, punctation similar to that of head, but with impunctate midline, interstices glossy.

Elytra wider than pronotum and nearly as long as pronotum (EL/EW = 0.95–1.0, EW/PW = 1.15–1.3, EL/PL = 0.91–0.94); punctation coarse and dense, arranged in series; interstices without microsculpture. Hind wings apparently present.

Abdomen broader than elytra; punctation fine and dense; posterior margin of tergite VII with palisade fringe.

**Male.** Sternite VII (Fig. 2D) strongly transverse and with distinct median impression, this impression without pubescence, posterior margin broadly concave; sternite VIII (Fig. 2E) weakly transverse, with long and pronounced postero-median impression, this impression with numerous modified, stout and short black setae, posterior excision moderately broad and moderately deep, on either side of this excision with long dark setae; aedeagus (Figs 2B, C) 1.70–1.72 mm long, ventral process long, flattened, and apically convex in ventral view.

**Female.** Unknown.

##### Etymology.

The specific epithet (Latin, adjective: of a duck) refers to the shape of the ventral process of the aedeagus, which somewhat resembles the mouth of a duckbill.

##### Comparative notes.

This species is close to *Lobrathium ablectum* Assing (2012) in sharing a similar shape and chaetotaxy of the sternites VII and VIII. The new species differs from *Lobrathium ablectum* by larger body size, and by the longer, apically not bifid ventral process of the aedeagus.

##### Habitat and distribution.

The present species was sifted from wet moss near a cold stream (Fig. 20A) in the Angjiangping National Reserve, Guangxi (Fig. 19), in July.

#### Lobrathium bispinosum

Assing

http://species-id.net/wiki/Lobrathium_bispinosum

Figs 3

Lobrathium bispinosum Assing, 2012: 97. Type locality: Leigong Shan, 15 km Leishan, Leishan County, Guizhou.

##### Material examined

(1 ♂)**. China, Jiangxi:** 1♂, Jinggangshan City, Ciping Town, 850 m, 26°29'N, 114°05'E, 18–X–2010, Peng et al. leg.

##### Distribution.

The original description of *Lobrathium bispinosum* is based on specimens from Guizhou (Assing 2012). The above record from Jiangxi extends the distributional range eastwards about 700 kilometers.

**Figure 3.**
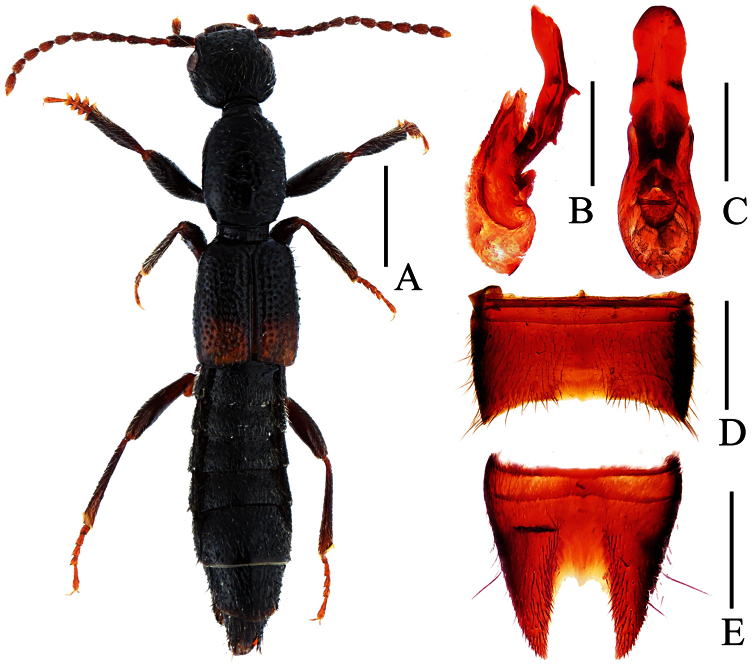
*Lobrathium bisponsum*. **A** habitus **B** aedeagus in lateral view **C** aedeagus in ventral view **D** male sternite VII **E** male sternite VIII. Scales: **A** 1mm, **B**–**E** 0.5mm.

#### Lobrathium configens

Assing

http://species-id.net/wiki/Lobrathium_configens

Figs 4

Lobrathium configens Assing, 2012: 93. Type locality: 115 km WSW Xi’an, river bank above Houzhenzi, Qinling Shan, Shaanxi.

##### Material examined

(2 ♂♂)**. China, Yunnan:** 1 ♂, Hutiaoxia, Jinxing, 1800 m, 27°11'N, 100°06'E, 22–IV–2005, Huang leg. **Qinghai:** 1 ♂, Menda N. R., 2500 m, 35°47'N, 107°48'E, 24–VII–2004, Hu et al. leg.

##### Distribution.

The original description of *Lobrathium configens* is based on specimens from Hubei, Sichuan, Yunnan, and Shaanxi (Assing 2012). The above records extend the wide distributional range of *Lobrathium configens* southwards by about 1200 kilometers.

**Figure 4.**
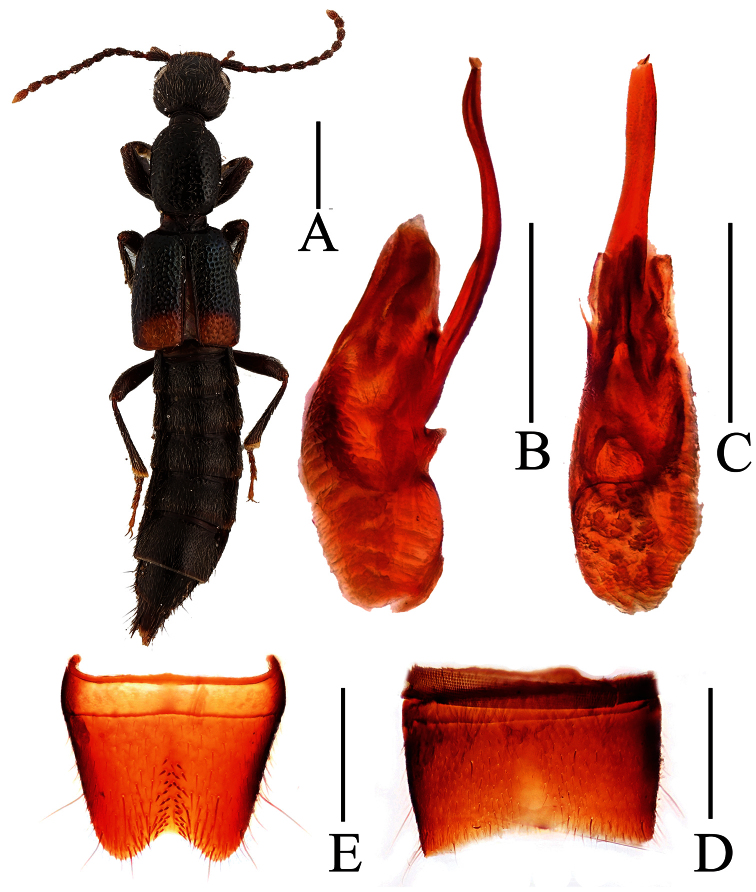
*Lobrathium configens*. **A** habitus **B** aedeagus in lateral view **C** aedeagus in ventral view **D** male sternite VII **E** male sternite VIII. Scales: **A** 1mm, **B**–**E** 0.5mm.

#### Lobrathium daxuense

Assing

http://species-id.net/wiki/Lobrathium_daxuense

Figs 5

Lobrathium daxuense Assing, 2012: 109. Type locality: Daxue Shan, Sichuan.

##### Material examined

(1 ♂)**. China, Sichuan:** 1 ♂, Tianquan County, Erlang Shan, Yakou 3.6 km, 29°31'N, 102°17'E, 2600–2800m, 11–VII–2012, Peng et al. leg.

##### Distribution.

Sichuan.

**Figure 5.**
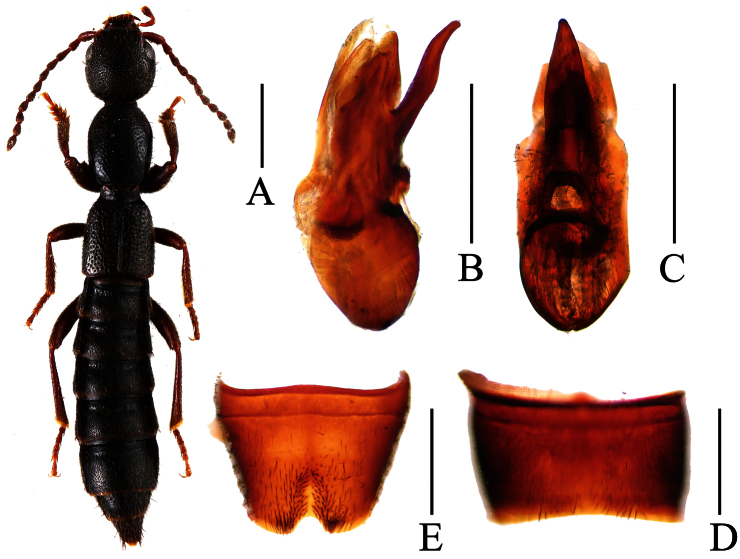
*Lobrathium daxuense*. **A** habitus **B** aedeagus in lateral view **C** aedeagus in ventral view **D** male sternite VII **E** male sternite VIII. Scales: **A** 1mm, **B**–**E** 0.5mm.

#### Lobrathium demptum

Assing

http://species-id.net/wiki/Lobrathium_demptum

Figs 6

Lobrathium demptum Assing, 2012: 97. Type locality: Dabie Shan, Hubei.

##### Material examined.

(47 ♂♂, 49 ♀♀)**. China, Zhejiang:** 17 ♂♂, 12 ♀♀, Anji County, Longwang Shan, 950–1200 m, 25–IV–2006, Shen et al. leg.; 8 ♂♂, 14 ♀♀, Anji County, Longwang Shan, 950–1200 m, 25–IV–2004, Tang et al. leg.; 2 ♂♂, 1 ♀, Anji County, Longwang Shan, 300–500 m, 23–IV–2004, Zhu leg.; 11 ♂♂, 12 ♀♀, Anji County, Longwang Shan, 03–X–2003, Hu et al. leg.; 8 ♂♂, 8 ♀♀, Lin’an City, East Tianmu Shan, 1050–1150 m, 13–IV–2011, Peng & Zhu leg.; 1 ♂, 2 ♀♀, Tianmu Shan, Gaoling, 800 m, 26–IV–2008, He & Tang leg.

##### Distribution.

The original description of *Lobrathium demptum* is based on specimens from the Dabie Shan, Hubei (Assing 2012). The above records from Zhejiang extend the range towards the southeast by about 400 kilometers.

**Figure 6.**
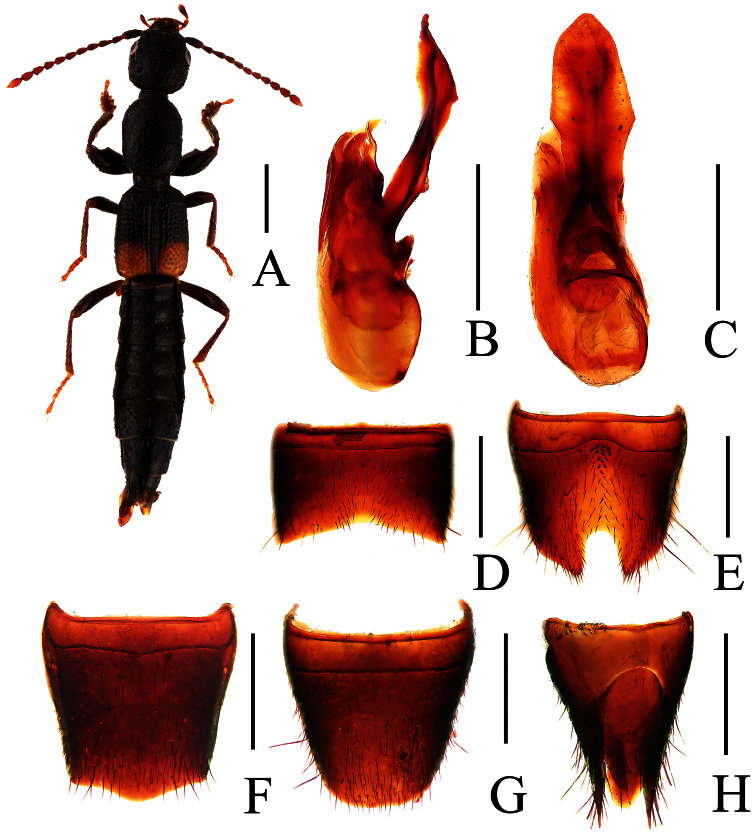
*Lobrathium demptum*. **A** habitus **B** aedeagus in lateral view **C** aedeagus in ventral view **D** male sternite VII **E** male sternite VIII **F** female tergite VIII **G** female sternite VIII **H** female tergites IX-X. Scales: **A** 1mm, **B**–**H** 0.5mm.

#### Lobrathium diaoluoense

Li & Li sp. n.

urn:lsid:zoobank.org:act:712D3A23-CF9D-454E-864A-48C10F88F56B

http://species-id.net/wiki/Lobrathium_diaoluoense

Figs 7

##### Type material

(10 ♂♂, 16 ♀♀)**. Holotype**, ♂: “China, Hainan, Lingshui County, Diaoluo Shan, 1000 m, 18°43'N, 109°51'E, 24–IV–2012, Peng Zhong & Dai Cong-chao leg. / Holotype ♂, *Lobrathium diaoluoense*, sp. n., Li & Li, det. 2013”. **Paratypes**, 9 ♂♂,16 ♀♀: same data as holotype.

##### Description.

**Figure 7.**
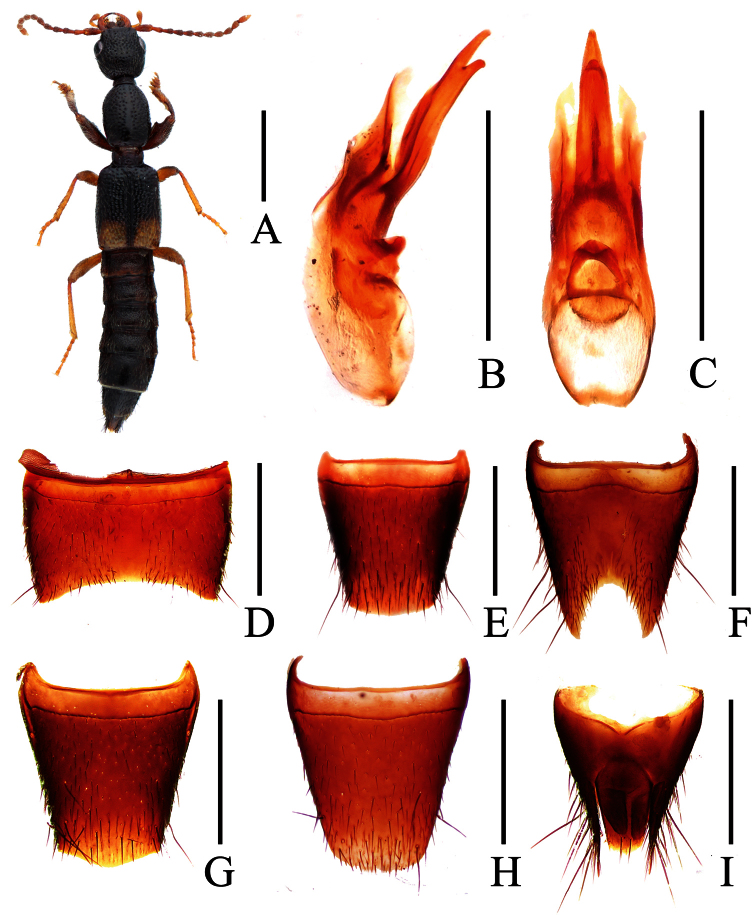
*Lobrathium diaoluoense*. **A** habitus **B** aedeagus in lateral view **C** aedeagus in ventral view **D** male sternite VII **E** male tergite VIII **F** male sternite VIII **G** female tergite VIII **H** female sternite VIII **I** female tergites IX–X. Scales: **A** 1mm, **B**–**I** 0.5mm.

Body length 4.61–5.95 mm, length of fore body 2.94–3.28 mm. Habitus as in Fig. 7A. Coloration: body black, posterior portion of elytra with yellowish spot reaching posterior and lateral margins; legs with paler tarsi; antennae yellowish.

Head as long as broad or weakly oblong (HL/HW = 1.0–1.09); posterior angles marked; punctation coarse and dense, sparser in median dorsal portion, interstices without microsculpture. Eyes large, more than half as long as distance from posterior margin of eye to neck. Antenna slender, 1.54–1.78 mm long.

Pronotum slender, approximately as wide as head (PL/PW = 1.25–1.31, PW/HW = 0.96–1.0), lateral margins weakly convex in dorsal view; punctation dense and coarse, similar to that of head, median dorsal portion more sparsely punctate or impunctate; interstices without microsculpture and glossy.

Elytra longer and broader than pronotum (EL/EW = 1.09–1.15, EW/PW = 1.24–1.37, EL/PL = 1.11–1.14); humeral angles marked; punctation coarse and dense, interstices without microsculpture and glossy. Hind wings fully developed.

Abdomen distinctly narrower than elytra; punctation fine and dense; posterior margin of tergite VII with palisade fringe; posterior margin of tergite VIII weakly concave, without appreciable sexual dimorphism.

**Male.** Sternite VII (Fig. 7D) with deep and broad median impression without pubescence, posterior margin broadly and rather strongly concave; sternite VIII oblong, with deep and large U-shaped posterior excision (Fig. 7F), without modified setae, on either side of posterior excision with long dark setae; aedeagus (Figs 7B, C) with ventral process of very distinctive morphology, furcate apically, and this bifurcation forming an angle of less than 30 degrees in lateral view.

**Female.** Posterior margin of tergite VIII (Fig. 7G) weakly convex; sternite VIII (Fig. 7H) of similar shape as tergite VIII; tergite IX (Fig. 7I) undivided anteriorly; tergite X of subovoid shape.

##### Etymology.

The specific epithet (adjective) is derived from the Diaoluo Shan where the type locality is situated.

##### Comparative notes.

This species is similar to *Lobrathium bipeniculatum* Assing (2010) and *Lobrathium pengi* Li & Li sp. n. (described below) in sharing similar shapes of the male sternites VII and VIII, and of the aedeagus. It can be separated from *Lobrathium bipeniculatum* by the broader median impression of the male sternite VII (Fig. 7D), and by the absence of clusters of long dark setae at the margins of the posterior excision of the male sternite VIII (Fig. 7F). In *Lobrathium pengi*, the ventral process of the aedeagus is of different shape, with the apical bifurcation forming an angle of more than 30 degrees in lateral view.

##### Habitat and distribution.

The present species was sifted from wet moss on stones alongside a reservoir (red circle in Fig. 20B) in the Diaoluo Shan, Hainan (Fig. 19), in April.

#### Lobrathium dufui

Li & Li sp.n.

urn:lsid:zoobank.org:act:48A1D1FD-6CAB-4238-8190-6FBB06DAB5B5

http://species-id.net/wiki/Lobrathium_dufui

Figs 8

##### Type material

(1 ♂, 2 ♀♀)**. Holotype**, ♂: “China, Hubei, Wufeng County, Houhe National Reserve, 30–IV–2004, 1100 m, 30°04'N, 110°37'E, Li Li-zhen leg. / Holotype ♂, *Lobrathium dufui*, sp. n. Li & Li, det. 2013”. **Paratypes**, 2 ♀♀: same data as holotype.

##### Description.

**Figure 8.**
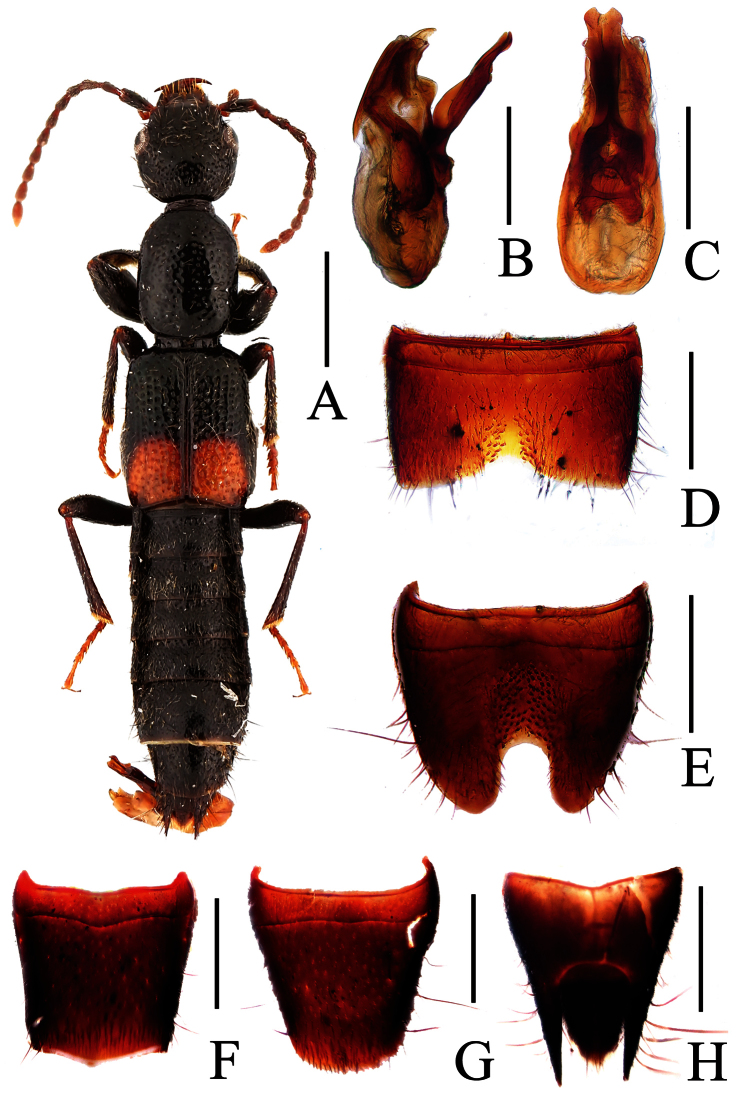
*Lobrathium dufui*. **A** habitus **B** aedeagus in lateral view **C** aedeagus in ventral view **D** male sternite VII **E** male sternite VIII **F** female tergite VIII **G** female sternite VIII **H** female tergites IX-X. Scales: **A** 1mm, **B**–**H** 0.5mm.

Body length 5.84–6.89 mm, length of fore body 3.34–3.61 mm. Habitus as in Fig. 8A. Coloration: body black, posterior portion of elytra with oblong yellowish spot of at least 2/5 the length of elytra and reaching posterior margins and lateral margins; legs blackish with slightly paler tibiae and tarsi; antennae brown.

Head weakly transverse (HW/HL = 1.02–1.09), widest at eyes, weakly tapering behind eyes; posterior angles rounded, not marked; punctation coarse and moderately dense, sparser in median dorsal portion and on frons; interstices without microsculpture. Eyes large, more than half the length of postocular region from posterior margin of eyes to neck in dorsal view. Antenna slender, 1.72–2.18 mm long.

Pronotum approximately as wide as head (PL/PW = 1.25–1.31, PW/HW = 0.96–1.10); lateral margins weakly convex in dorsal view; punctation dense and coarse, similar to that of head, but with impunctate midline; interstices without microsculpture and glossy.

Elytra broader than pronotum (EL/EW = 1.02–1.06, EW/PW = 1.21–1.39, EL/PL = 1.01–1.08); humeral angles marked; punctation coarse and dense. Hind wings apparently present.

Abdomen distinctly narrower than elytra; punctation fine and dense; posterior margin of tergite VII with palisade fringe; tergite VIII (Fig. 8F) without appreciable sexual dimorphism, posterior margin broadly convex.

**Male.** sternite VII (Fig. 8D) transverse and posteriorly with pronounced impression of triangular shape, this impression impunctate in the middle and laterally with a few modified, stout and short black setae, posterior margin broadly and weakly concave; sternite VIII (Fig. 8E) weakly transverse, with deep and broad, U-shaped posterior excision, median impression furnished with numerous modified, stout, short and black setae; aedeagus (Figs 8B, C) with ventral process of very distinctive morphology, apically with fissure and bifid.

**Female.** Sternite VIII (Fig. 8G) weakly transverse, posteriorly convex; tergite IX (Fig. 8H) undivided anteriorly, anterior margin emarginated in the middle; tergite X of subovoid shape.

##### Etymology.

The species is named after the famous late poet Fu Du, who was born in Hubei.

##### Comparative notes.

This species is similar to *Lobrathium uncinatum* Li & Li sp. n. (described below) in external characters, and to *Lobrathium hebeatum* Zheng (1988) in sexual characters. It differs from *Lobrathium uncinatum* by the shape of the aedeagus and by the absence of modified setae on the male sternite VI, and from *Lobrathium hebeatum* by the shape of the apex of the ventral process of the aedeagus.

##### Habitat and distribution.

The type specimens were sifted from wet moss near a stream (Fig. 20C) in Hubei (Fig. 19).

#### Lobrathium hebeatum

Zheng

http://species-id.net/wiki/Lobrathium_hebeatum

Figs 9

Lobrathium hebeatum Zheng, 1988: 189. Type locality: Emei Shan, Sichuan.
Lobrathium hebeatum Zheng: Assing, 2012: 91. New distribution: Sichuan, Shaanxi, Yunnan.

##### Material examined

(6 ♂♂, 8 ♀♀)**. China, Shaanxi:** 4 ♂♂, 5 ♀♀, Foping N. R., 1400–1800 m, 33°31'N, 107°56'E, 19–VII–2004, Hu et al. leg.; 1 ♂,1 ♀, Taibai Shan, 1450–1750 m, 34°03'N, 107°53'E – 33°53'N, 107°48'E, 15–VII–2004, Hu & Tang leg. **Sichuan:** 1 ♂,2 ♀♀, Luding County, Hailuogou, 3000 m, 29°44'N, 102°07'E, 25–VII–2006, Hu & Tang leg.

##### Distribution.

Sichuan, Yunnan, Shaanxi.

**Figure 9.**
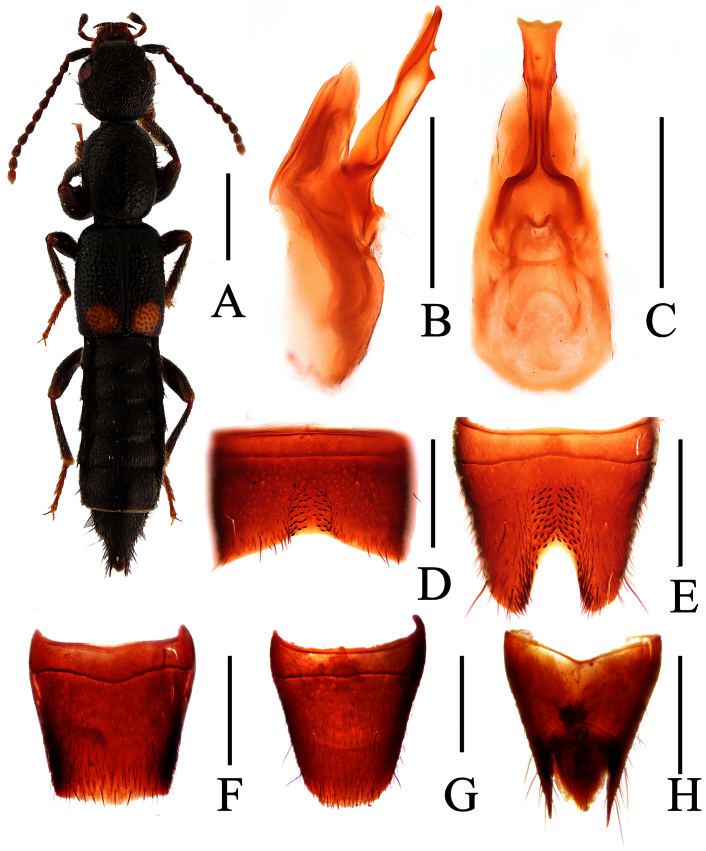
*Lobrathium hebeatum*. **A** habitus **B** aedeagus in lateral view **C** aedeagus in ventral view **D** male sternite VII **E** male sternite VIII **F** female tergite VIII **G** female sternite VIII **H** female tergites IX-X. Scales: **A** 1mm, **B**–**H** 0.5mm.

#### Lobrathium hongkongense

Bernhauer

http://species-id.net/wiki/Lobrathium_hongkongense

Figs 10

Lathrobium hongkongense Bernhauer, 1931: 127. Type locality: Hongkong.
Lobrathium sibynium Zheng, 1988: 186. Distribution: Sichuan; Assing, 2012: 86, proposed synonymy. New distribution: Jiangsu, Zhejiang, Hubei, Guangxi, Sichuan, Yunnnan.

##### Material examined

(67 ♂♂, 84 ♀♀)**. China, Fujian:** 1 ♂, 1 ♀, Guihe Village, Meihua Shan, 1200 m, 25°19'N, 116°51'E, 31–V–2007, Huang & Xu leg. **Guizhou:** 1 ♂, 1 ♀, Suiyang County, Kuankuoshui N. R., Baishaogou, 700 m, 28°10'N, 107°16'E, 04–VI–2010, Lu et al. leg. **Yunnan:** 3 ♂♂, 1 ♀, Nabanhe N. R., Chuguohe, Bengganghani, 1750 m, 28–IV–2009, Hu & Yin leg. **Zhejiang:** 1 ♂, 2 ♀♀, Zhuji City, Dongbai Shan, 200 m, 29°31'N, 120°25'E, 06–V–2012, Zhao leg.; 8 ♂♂, 12 ♀♀, Wuyanling N. R., 700 m, 10–IV–2004, Hu et al. leg.; 7 ♂♂, 13 ♀♀,Wenzhou City, Yandang Shan, 50–350 m, 29–V–2006, Li & Shen leg.; 1 ♂, 1 ♀, Pan’an County, Dapan Shan, 550–800 m, 06–VI–2006, Li & Shen leg.; 25 ♂♂, 22 ♀♀, Baishanzu N.R., 1200–1360 m, 05–V–2004, Hu et al. leg.; 20 ♂♂, 31 ♀♀, Gutian N. R., 5~7–V–2005, Zhu & Li leg.

##### Comment.

**Figure 10.**
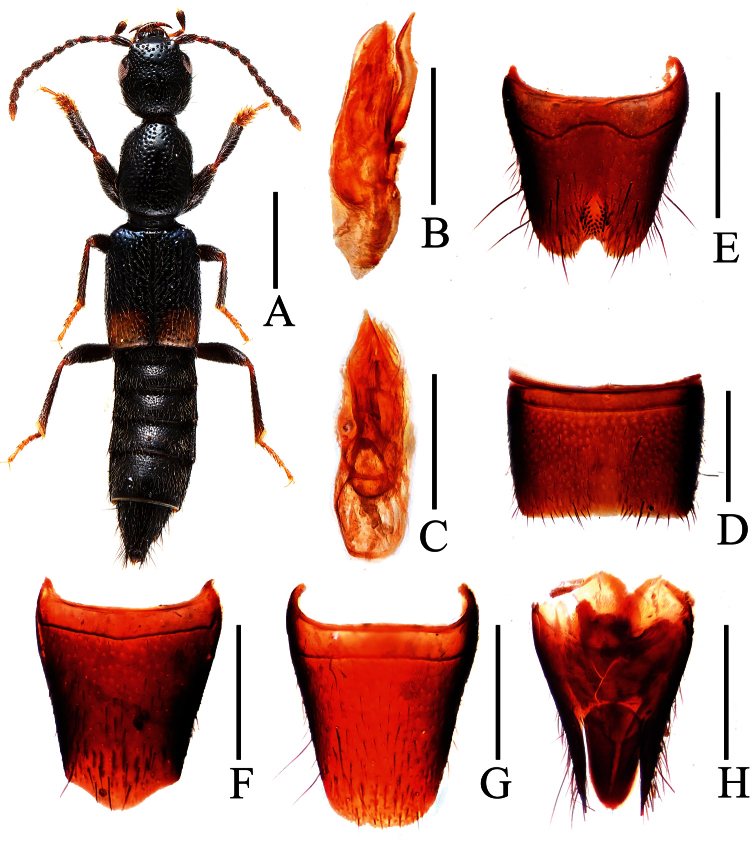
*Lobrathium hongkongense*. **A** habitus **B** aedeagus in lateral view **C** aedeagus in ventral view **D** male sternite VII **E** male sternite VIII **F** female tergite VIII **G** female sternite VIII **H** female tergites IX–X. Scales: **A** 1mm, **B**–**H** 0.5mm.

For illustrations of the female sexual characters see Figs 10G–H.

##### Distribution.

Widespread species, recorded from Jiangsu, Zhejiang, Fujian, Hubei, Guangxi, Sichuan, Guizhou, and Yunnan.

#### Lobrathium lirunyui

Li & Li sp. n.

urn:lsid:zoobank.org:act:BD413210-58A8-4AF0-88F7-F159C30D5E3E

http://species-id.net/wiki/Lobrathium_lirunyui

Figs 11

##### Type material

(1 ♂)**. Holotype**, ♂: “China, Guizhou, Zunyi City, Fenghuang Shan, 800 m, 27°41'N, 106°55'E, 19–VI–2012, Li Run-yu leg. / Holotype ♂, *Lobrathium lirunyui*, sp. n. Li & Li, det. 2013”.

##### Description.

**Figure 11.**
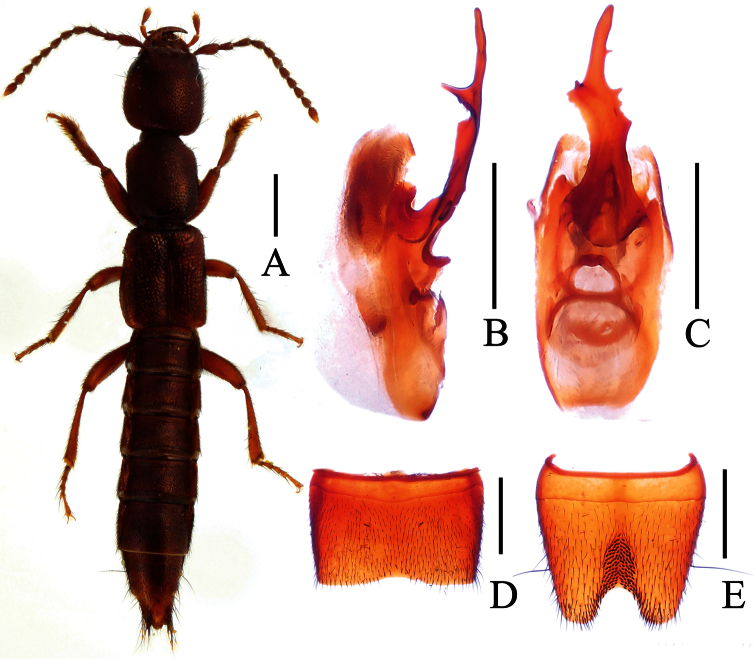
*Lobrathium lirunyui*. **A** habitus **B** aedeagus in lateral view **C** aedeagus in ventral view **D** male sternite VII **E** male sternite VIII. Scales: **A** 1mm, **B**–**E** 0.5mm.

Large species, body length 9.40 mm, length of fore body 4.20 mm. Habitus as in Fig. 11A. Body reddish brown, legs reddish with pale-reddish tarsi, antennae reddish to brown.

Head longer than wide (HL/HW = 1.06), widest posteriorly; posterior angles weakly marked; punctation of dorsal surface fine and very dense; interstices without microsculpture. Eyes small, approximately one third the length of distance from posterior margin of eye to neck in dorsal view. Antenna long and slender, 2.50 mm long.

Pronotum slender (PL/PW = 1.28, PW/HW = 0.90), lateral margins almost straight and subparallel in dorsal view; punctation similar to that of head, but with impunctate midline.

Elytra longer than wide (EL/EW = 1.02, EW/PW = 1.25, EL/PL = 1.08); punctation coarse and dense, arranged in somewhat irregular series; interstices without microsculpture. Hind wings apparently fully developed.

Abdomen distinctly narrower than elytra; punctation fine and dense; posterior margin of tergite VII with palisade fringe.

**Male.** Sternite VII (Fig. 11D) strongly transverse, and with shallow median impression posteriorly, without modified setae, posterior margin broadly and weakly concave; sternite VIII (Fig. 11E) weakly transverse, with long and extensive postero-median impression, this impression with numerous modified, stout and short black setae, posterior excision rather broad and U-shaped, on either side of this excision with long dark submarginal setae; aedeagus (Figs 11B, C) 1.56 mm long, with asymmetric ventral process of distinctive shape.

**Female.** Unknown.

##### Etymology.

The species is named after Runyu Li, collector of the holotype.

##### Comparative notes.

This species is readily distinguished from all its congeners by the following character combination: elytra without spot, whole body of brownish coloration; punctation of head fine and dense, eyes very small, one third as long as distance from posterior margin of eye to neck, male sexual characters highly distinctive.

##### Habitat and distribution.

The holotype was sifted in fern vegetation near a wet tree root (Fig. 20D) in the Fenghuang Shan, Guizhou (Fig. 19).

#### Lobrathium pengi

Li & Li sp. n.

urn:lsid:zoobank.org:act:50DF2D82-E0AD-4B81-996D-BEFC980CD79A

http://species-id.net/wiki/Lobrathium_pengi

Figs 12

##### Type material

(7 ♂♂, 3 ♀♀)**. Holotype**, ♂: “China, Guangxi, Shangsi County, Shiwanda Shan, Forest Park, 300–500 m, 21°54'N, 107°54'E, 25–IV–2011, Peng Zhong & Zhu Jian-qing leg. / Holotype ♂, *Lobrathium pengi*, sp. n. Li & Li, det. 2013”. **Paratypes**, 6 ♂♂, 3♀♀: same data as holotype.

##### Description.

**Figure 12.**
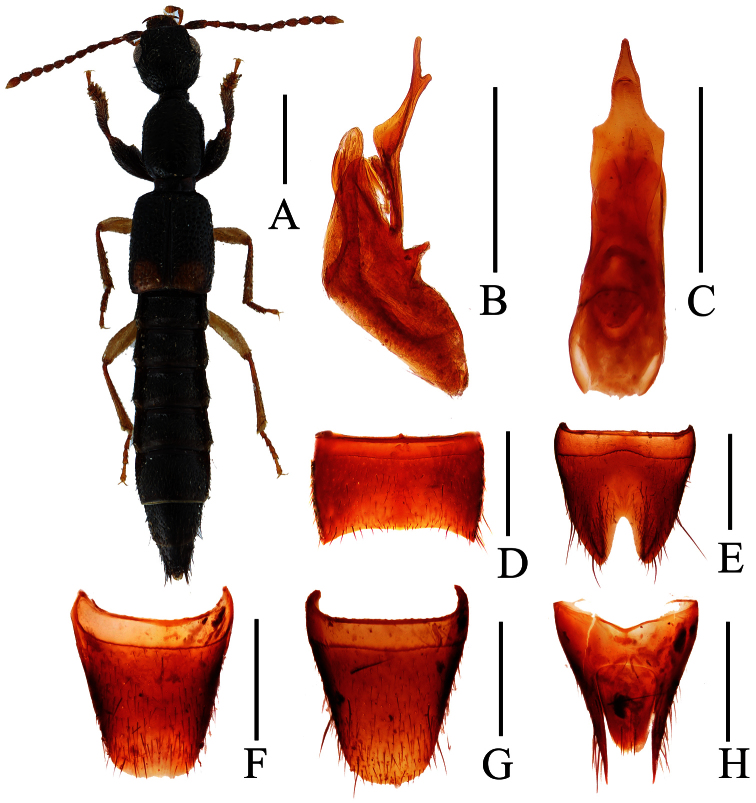
*Lobrathium pengi*. **A** habitus **B** aedeagus in lateral view **C** aedeagus in ventral view **D** male sternite VII **E** male sternite VIII **F** female tergite VIII **G** female sternite VIII **H** female tergites IX-X. Scales: **A** 1mm, **B**–**H** 0.5mm.

Body length 6.34–7.34 mm, length of fore body 3.11–3.73 mm. Habitus as in Fig. 12A. Coloration: body black, elytra in posterior 1/3–2/5 with subcircular yellowish spot reaching posterior, but not lateral margins; legs yellowish with forelegs brown to blackish, femora and protibiae and tarsi paler; antennae brown.

Head weakly transverse (HW/HL = 1.02–1.03); posterior angles not marked; punctation coarse and dense, sparser in median dorsal portion, interstices without microsculpture. Eyes large, more than half as long as the distance from posterior margin of eye to neck. Antenna slender, 1.68–1.82 mm long.

Pronotum nearly as wide as head (PL/PW = 1.13–1.33, PW/HW = 0.97–0.98), lateral margins weakly convex in dorsal view; punctation dense and coarse, similar to that of head, but with impunctate midline; interstices without microsculpture and glossy.

Elytra broader than pronotum (EL/EW = 1.0–1.06, EW/PW = 1.35–1.40, EL/PL = 0.98–1.12); humeral angles marked; punctation coarse and dense. Hind wings fully developed.

Abdomen distinctly narrower than elytra; punctation fine and dense; posterior margin of tergite VII with palisade fringe; tergite VIII (Fig. 12F) without appreciable sexual dimorphism, with weakly convex posterior margin.

**Male.** Sternite VII (Fig. 12D) strongly transverse, posterior impression of triangular shape and impunctate, posterior margin broadly and concave; sternite VIII (Fig. 12E) weakly transverse, without modified setae, posterior excision deep and rather narrow, on either side of this excision with long dark setae; aedeagus (Fig. 12B, C) approximately 1.0 mm long, ventral process of very distinctive morphology, slender and furcate apically, bifurcation forming an angle of more than 30 degrees in lateral view.

**Female.** Sternite VIII as in Fig. 12G; tergites IX-X (Fig. 6H) relatively short; tergite IX undivided anteriorly, anterior martin emarginated in the middle; tergite X of subovoid shape.

##### Etymology.

The species is named after Zhong Peng, collector of the type specimens.

##### Comparative notes.

This species is similar to *Lobrathium diaoluoense*, from which it is separated by the broader and apically more abruptly narrowed ventral process of the aedeagus, with the apical bifurcation forming an angle of more than 30 degrees in lateral view.

##### Habitat and distribution.

The specimens were sifted from wet moss along a streamside (Fig. 20E) in the Shiwanda Shan, Guangxi (Fig. 19), in April.

#### Lobrathium quyuani

Li & Li sp. n.

urn:lsid:zoobank.org:act:B2CB6E5C-8ECB-453C-BB8C-1DA7F5969833

http://species-id.net/wiki/Lobrathium_quyuani

Figs 13

##### Material

(3 ♂♂)**. Holotype**, ♂: “China, Hubei, Wufeng County, Houhe National Reserve, 1100 m, 30°04'N, 110°37'E, 30–IV–2004, Li Li-zhen leg. / Holotype ♂, *Lobrathium quyuani*, sp. n. Li & Li, det. 2013”. **Paratypes**, 2 ♂♂: same data as holotype.

##### Description.

**Figure 13.**
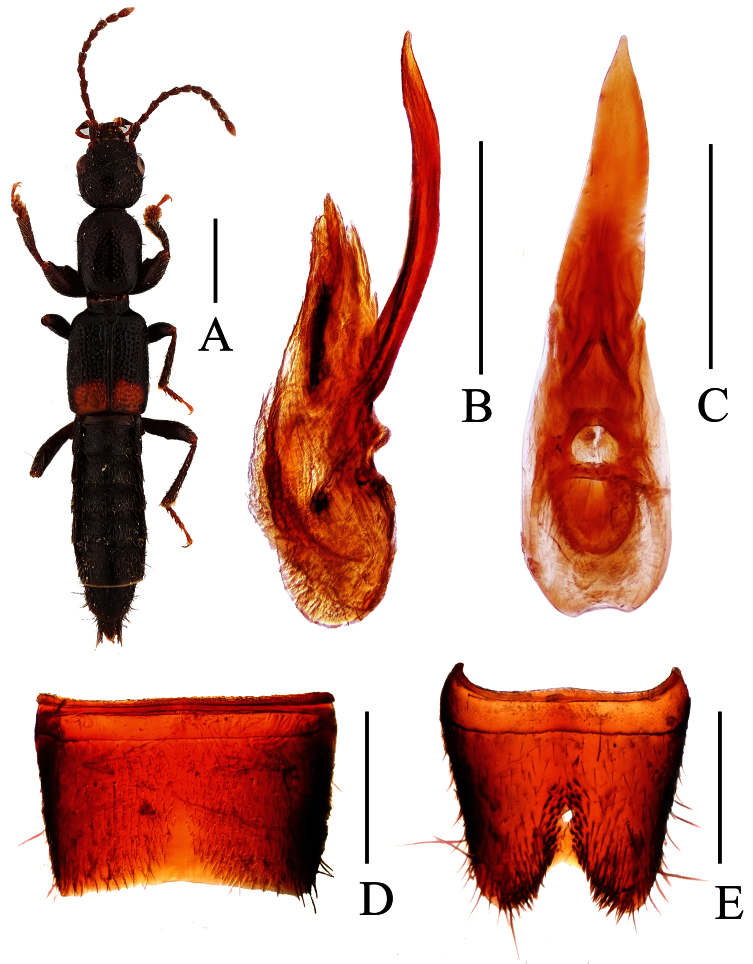
*Lobrathium quyuani*. **A** habitus **B** aedeagus in lateral view **C** aedeagus in ventral view **D** male sternite VII **E** male sternite VIII. Scales: **A** 1mm, **B**–**E** 0.5mm.

Body length 5.78–6.34 mm, length of fore body 3.28–3.56 mm. Habitus as in Fig. 13A. Coloration: body black with bluish hue, elytra with yellowish to reddish spot reaching posterior but not lateral margins; legs blackish with dark-brownish tarsi; antennae dark-brownish.

Head as wide as long (HW/HL = 0.96–1.03), widest at eyes; posterior angles broadly rounded; punctation dense and moderately coarse, sparser in median dorsal portion; interstices without microsculpture. Eyes large, more than half as long as the distance from posterior margin of eye to neck in dorsal view. Antenna slender, 1.72–1.95 mm long.

Pronotum slender (PL/PW = 1.25–1.41, PW/HW= 0.92–1.0), lateral margins weakly convex in dorsal view, with impunctate midline, punctation similar to that of head, but distinctly sparser, interstices glossy.

Elytra longer than broad (EL/EW = 1.0–1.02, EW/PW = 1.24–1.33, EL/PL = 1.0–1.05); punctation coarse and dense, arranged in irregular series; interstices without microsculpture. Hind wings fully developed.

Abdomen narrower than elytra; punctation fine and dense; posterior margin of tergite VII with palisade fringe; posterior margin of tergite VIII strongly convex in the middle.

**Male.** Sternite VII (Fig. 13D) strongly transverse, posteriorly with pronounced median impression, this impression of somewhat triangular shape, without punctation and pubescence, posterior margin broadly and very weakly concave; sternite VIII (Fig. 3E) weakly transverse, postero-median impression with modified, stout and short black setae, posterior excision deep and moderately broad, on either side of this excision with long dark submarginal setae; aedeagus (Figs 13B, C) 1.36 mm long, with weakly asymmetric and apically acute ventral process.

**Female.** Unknown.

##### Etymology.

The specific epithet refers to the famous late poet Yuan Qu, who was born in Yichang (Hubei), which is near the type locality.

##### Comparative notes.

This species is similar to *Lobrathium configens* Assing (2012) and *Lobrathium spathulatum* Assing (2012) in external characters. It is distinguished from both by the broader ventral process in ventral view.

##### Habitat and distribution.

The specimens were sifted from wet moss near a stream (Fig. 20C) in the Houhe National Reserve, Hubei (Fig. 19).

#### Lobrathium spathulatum

Assing

http://species-id.net/wiki/Lobrathium_spathulatum

Figs 14

Lobrathium spathulatum Assing, 2012: 95. Type locality: Pingwu, Sichuan.

##### Material examined

(2 ♂♂, 2 ♀♀)**. China, Zhejiang:** 1 ♂, 1 ♀, Anji County, Longwang Shan, Pingxi, 1000–1100 m, 09–VI–2012, Hu & Yin leg.; 1 ♂, 1 ♀, Qingliangfeng, 1050–1070 m, 09–V–2005, Zhu & Li leg.

##### Distribution.

Widespread, recorded from Hubei, Shanxi, Zhejiang, Sichuan, and Shaanxi.

**Figure 14.**
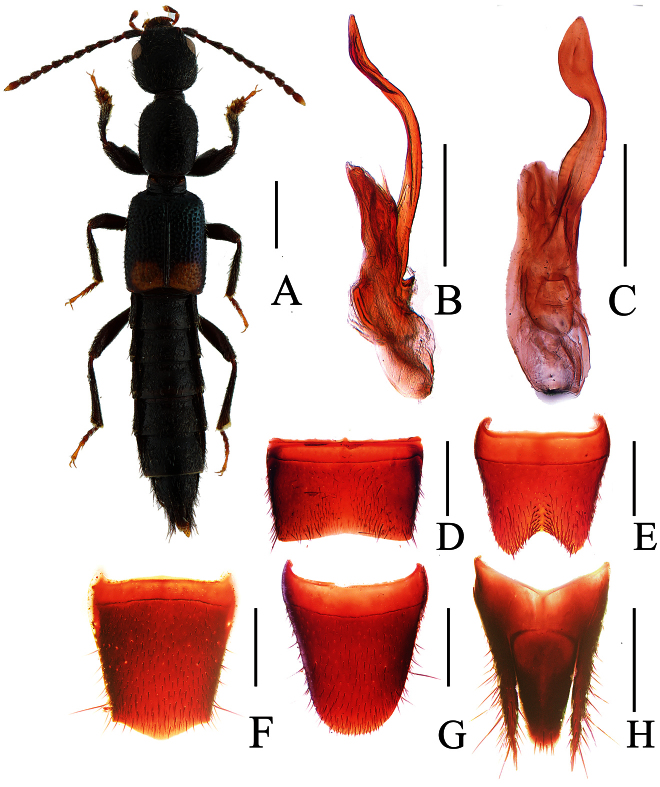
*Lobrathium spathulatum*. **A** habitus **B** aedeagus in lateral view **C** aedeagus in ventral view **D** male sternite VII **E** male sternite VIII **F** female tergite VIII **G** female sternite VIII **H** female tergites IX-X. Scales: **A** 1mm, **B**–**H** 0.5mm.

#### Lobrathium taureum

Assing

http://species-id.net/wiki/Lobrathium_taureum

Figs 15

Lobrathium taureum Assing, 2012: 100. Type locality: creek valley 8 km NW Muyuping, Daba Shan, Hubei.

##### Material examined

(1 ♂)**. China, Shanxi:** 1 ♂, Ningwu County, Ximafang, 1430m, 38°39'N, 112°01'E, 04–IX–2011, Peng leg.

##### Distribution.

Beijing, Hubei, Shanxi.

**Figure 15.**
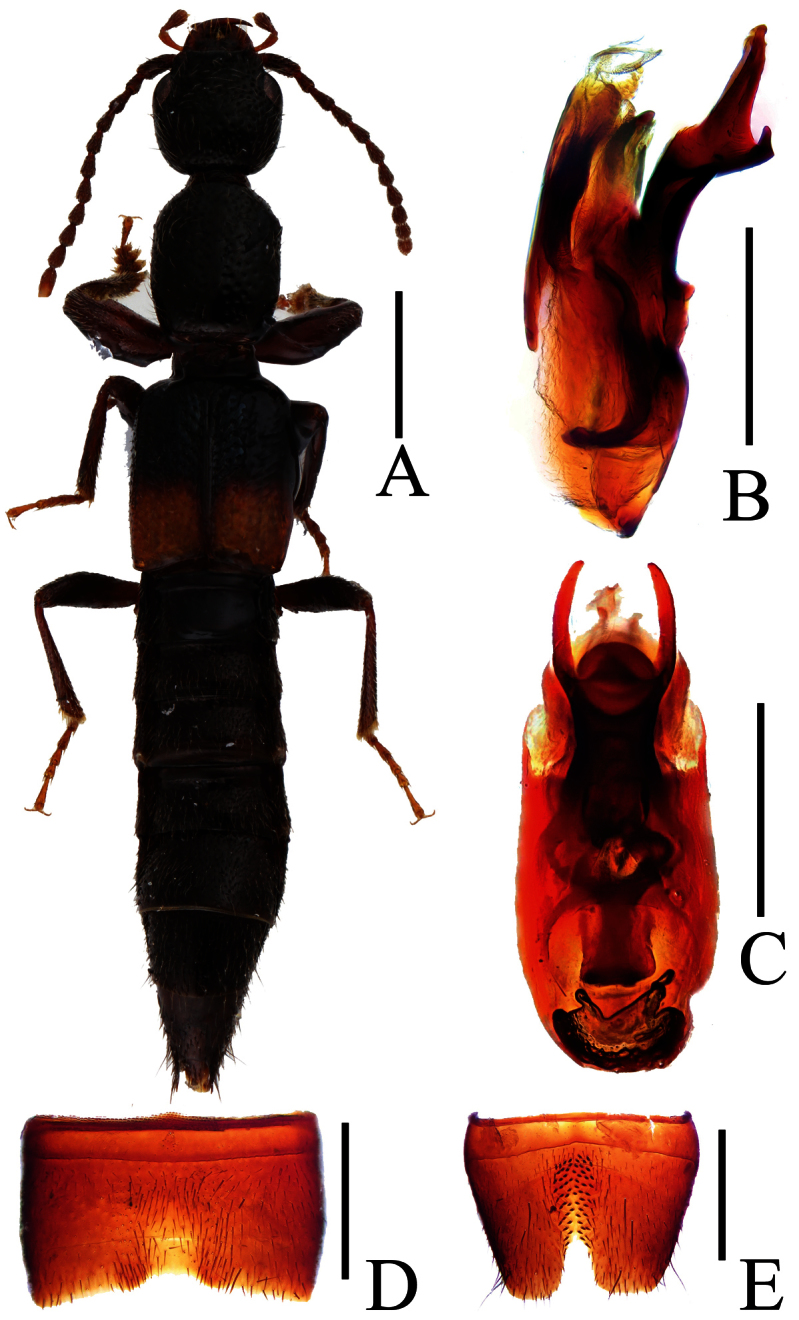
*Lobrathium taureum*. **A** habitus **B** aedeagus in lateral view **C** aedeagus in ventral view **D** male sternite VII **E** male sternite VIII. Scales: **A** 1mm, **B**–**E** 0.5mm.

#### Lobrathium tortile

Zheng

http://species-id.net/wiki/Lobrathium_tortile

Figs 16

Lobrathium tortile Zheng, 1988: 187. Type locality: Kangding, Sichuan.
Lobrathium tortile Zheng: Assing, 2012: 89. New distribution: Hubei, Sichuan, Shaanxi.

##### Additional material examined

(6 ♂♂, 3 ♀♀)**. China, Shaanxi:** 1 ♂, Taibai Shan, 1450–1750 m, 34°03'N, 107°53'E – 33°53'N, 107°48'E, 15–VII–2004, Hu & Tang leg.; 3 ♂♂, 2 ♀♀, Zhouzhi County, Houzhenzi, Qinling Shan, West Sangongli Valley, 33°50'N, 107°48'E, 17~19–V–2008, Huang & Xu leg. **Guizhou:** 1 ♂, Suiyang County, Kuankuoshui N. R., Baishaogou, 700 m, 28°10'N, 107°16'E, 03–VI–2010, Lu et al. leg. **Sichuan:** 1 ♂, 1♀, Shimian County, Liziping, 1800 m, 28°59'N, 102°28'E, 16–VII–2012, Dai et al. leg.

##### Distribution.

Sichuan, Shaanxi, Hubei, Guizhou.

**Figure 16.**
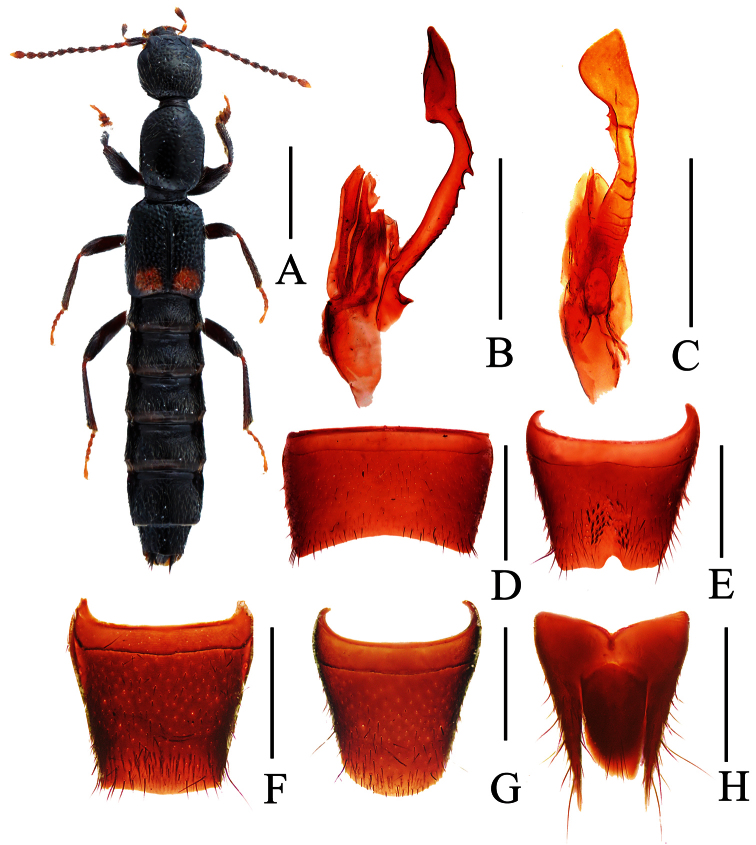
*Lobrathium tortile*. **A** habitus **B** aedeagus in lateral view **C** aedeagus in ventral view **D** male sternite VII **E** male sternite VIII **F** female tergite VIII **G** female sternite VIII **H** female tergites IX-X. Scales: **A** 1mm, **B**–**H** 0.5mm.

#### Lobrathium tortuosum

Li et al.

http://species-id.net/wiki/Lobrathium_tortuosum

Figs 17

Lobrathium tortuosum Li, Solodovnikov & Zhou, 2013: 574. Type locality: Fengyang Shan, Zhejiang.

##### Material examined

(3 ♂♂, 1♀)**. China, Zhejiang:** 1 ♂, Qingliangfeng, 1080 m, 08–V–2005, Zhu & Li leg.; 1 ♂, 1 ♀, Longquan City, Fengyang Shan, 1450–1600 m, 22–VII–2006, Shen & Li leg.; 1 ♂, Baishanzu N.R., 1200–1360 m, 05–V–2004, Hu et al. leg.

##### Distribution.

The original description of *Lobrathium tortuosum* is based on specimens from the Fengyang Shan, Zhejiang (Li et al., 2013). The above records from Qingliangfeng extend the distributional range about 200 km northwards. The known distribution is confined to the Tianmu Shan and the Donggong Shan in Zhejiang.

**Figure 17.**
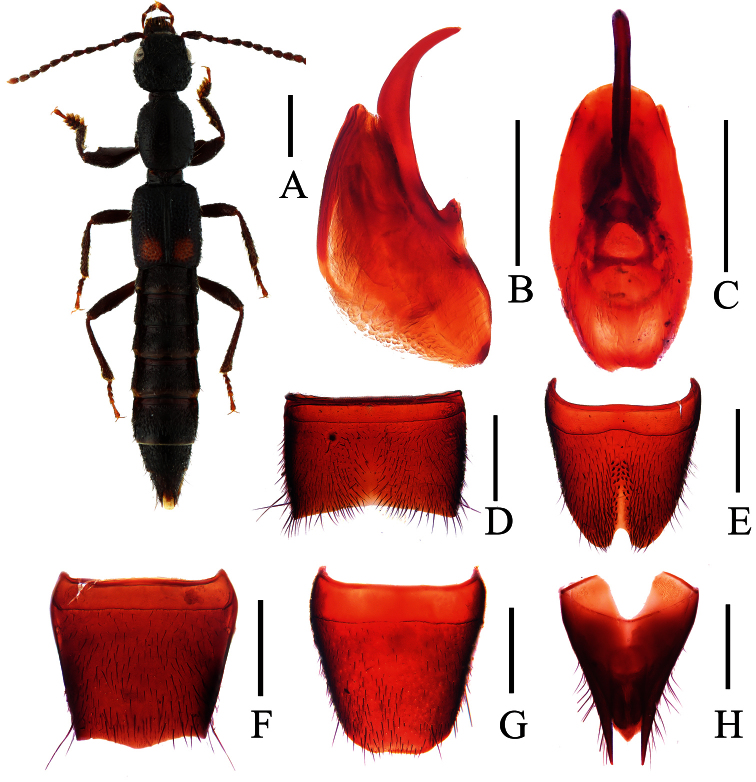
*Lobrathium tortuosum*. **A** habitus **B** aedeagus in lateral view **C** aedeagus in ventral view **D** male sternite VII **E** male sternite VIII **F** female tergite VIII **G** female sternite VIII **H** female tergites IX-X. Scales: **A** 1mm, **B**–**H** 0.5mm.

#### Lobrathium uncinatum

Li & Li sp. n.

urn:lsid:zoobank.org:act:A321CDF5-7B72-4699-BB65-C42C5C924BDF

http://species-id.net/wiki/Lobrathium_uncinatum

Figs 18A–F

##### Type material

(1 ♂)**. Holotype**, ♂: “China, Qinghai, Xining City, Huzhu County, Beishan National Reserve, 2450 m, 36°56'N, 102°27'E, 28–VII–2004, Tang Liang, Hu Jia-yao & Zhu Li-long leg. / Holotype ♂, *Lobrathium uncinatum*, sp. n., Li & Li, det. 2013”.

##### Description.

**Figure 18.**
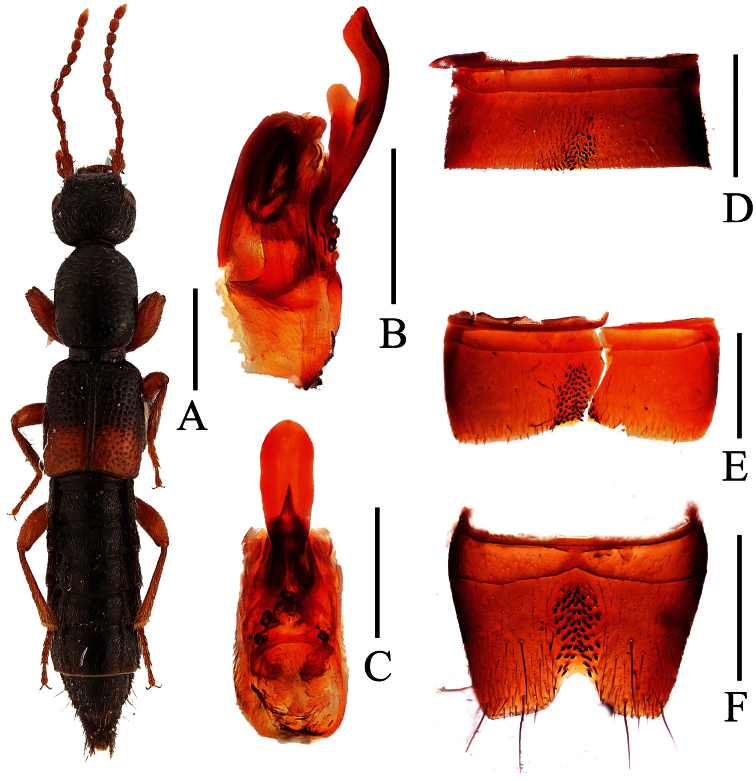
*Lobrathium uncinatum*. **A** habitus **B** aedeagus in lateral view **C** aedeagus in ventral view **D**  male sternite VI **E** male tergite VII **F** male sternite VIII. Scales: **A** 1mm, **B**–**F** 0.5mm.

Body length 5.94 mm, length of fore body 3.16 mm. Habitus as in Fig. 18A. Coloration: body black, elytra posteriorly with large yellowish spot reaching posterior and lateral margins; legs reddish with paler tarsi; antennae reddish.

Head transverse (HW/HL = 1.14); posterior angles not marked; punctation coarse and dense, sparser in median dorsal portion, interstices without microsculpture. Eyes large, more than half as long as distance from posterior margin of eye to neck. Antenna slender, 1.92 mm long.

Pronotum moderately oblong, as wide as head (PL/PW = 1.18, PW/HW = 1.0); lateral margins subparallel in dorsal view; punctation dense and coarse, similar to that of head, but with impunctate midline; interstices without microsculpture and glossy.

Elytra longer than broad, broader than pronotum (EL/EW = 1.0, EW/PW = 1.29, EL/PL = 0.98); humeral angles marked; punctation coarse and dense. Hind wings fully developed.

Abdomen narrower than elytra; punctation fine and dense; posterior margin of tergite VII with palisade fringe.

**Male.** Sternite VI (Fig. 18D) strongly transverse, postero-medially with modified stout black setae; sternite VII (Fig. 18E) strongly transverse, posteriorly with pronounced impression, this impression with numerous modified stout black setae, posterior margin broadly and weakly concave; sternite VIII (Fig. 18F) transverse, postero-median impression with modified setae like on sternites VI and VII, posteriorly with moderately deep excision; aedeagus (Figs 18B, C) with ventral process of distinctive morphology, near middle with distinct dorsal projection in lateral view.

**Female.** Unknown.

##### Etymology.

The specific epithet (Latin, hooked) refers to the shape of the ventral process of the aedeagus.

##### Comparative notes.

This species can be separated from the externally similar *Lobrathium taureum* Assing (2012) and *Lobrathium schuelkei* Assing (2012) by the presence of modified setae on the male sternites VI and VII (Figs 18D, E) and by the distinctive shape of the ventral process of the aedeagus.

##### Habitat and distribution.

**Figure 19.**
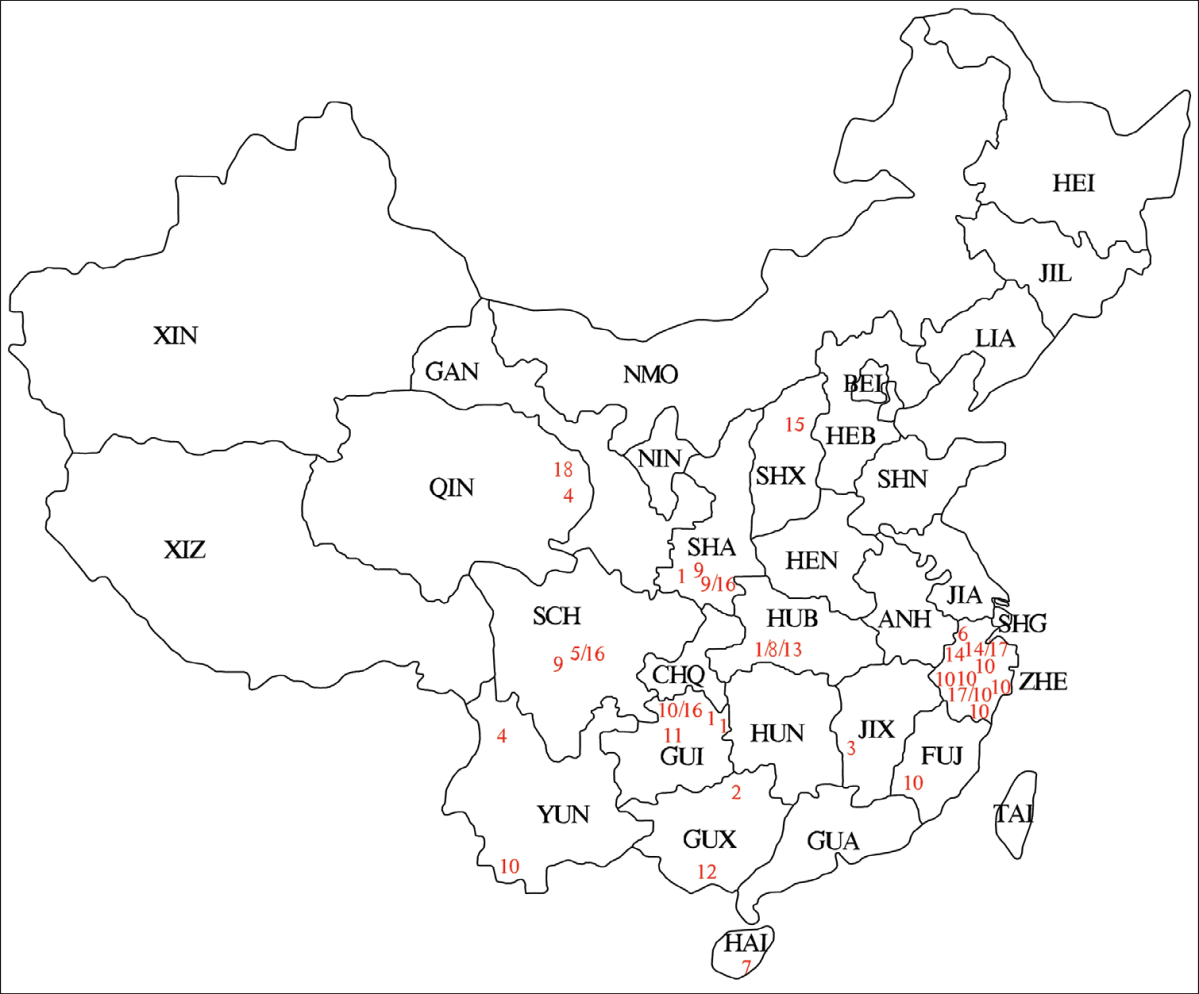
Map of collecting sites. **1** *Lobrathium ablectum* **2** *Lobrathium anatitum* **3** *Lobrathium bispinosum* **4** *Lobrathium configens* **5** *Lobrathium daxuense* **6** *Lobrathium demptum* **7** *Lobrathium diaoluoense* **8** *Lobrathium dufui* **9** *Lobrathium hebeatum* **10** *Lobrathium hongkongense* **11** *Lobrathium lirunyui* **12** *Lobrathium pengi* **13** *Lobrathium quyuani* **14** *Lobrathium spathulatum* **15** *Lobrathium taureum* **16** *Lobrathium tortile* **17** *Lobrathium tortuosum* **18** *Lobrathium uncinatum*. ANH Anhui; BEI Beijing; CHQ Chongqing; FUJ Fujian; GAN Gansu; GUA Guangdong; GUI Guizhou; GUX Guangxi; HAI Hainan; HEB Hebei; HEI Heilongjiang; HEN Henan; HUB Hubei; HUN Hunan; JIA Jiangsu; JIL Jilin; JIX Jiangxi; LIA Liaoning; NIN Ningxia; NMO Nei Mongol; QIN Qinghai; SCH Sichuan; SHA Shaanxi; SHG Shanghai; SHX Shanxi; SHN Shandong; TAI Taiwan; XIN Xinjiang; XIZ Xizang; YUN Yunnan; ZHE Zhejiang.

The holotype was sifted from wet moss alongside a river bank (Fig. 20F) in the Meda National Reserve, Qinghai (Fig. 19).

**Firure 20.**
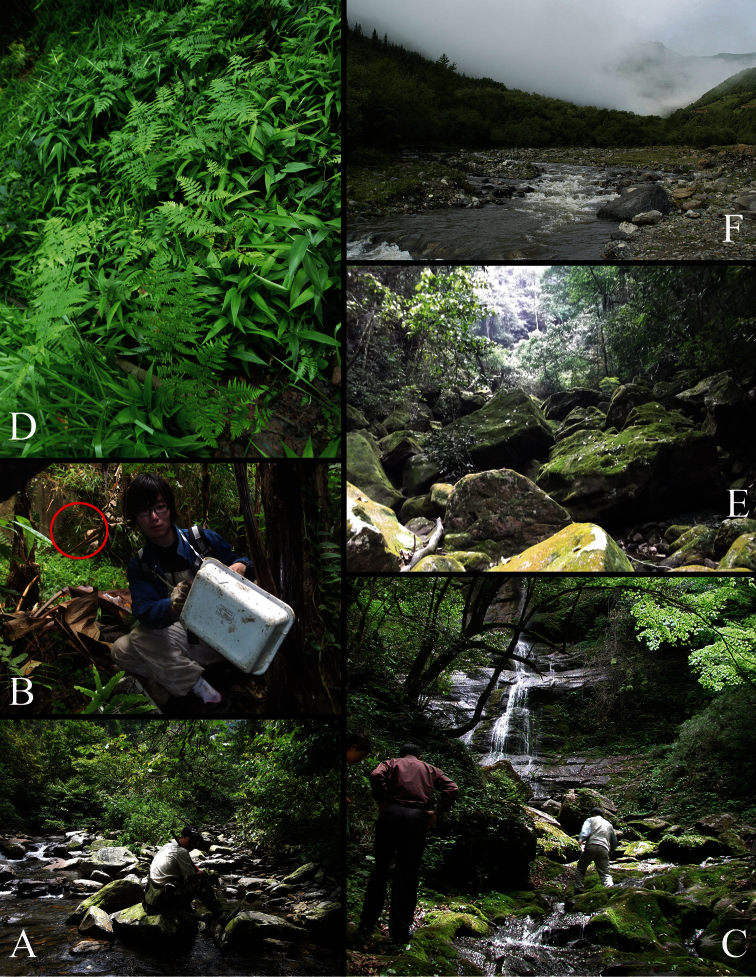
Habitat of new species. **A** *Lobrathium anatitum* **B** *Lobrathium diaoluoense* **C** *Lobrathium dufui*; *Lobrathium quyuani* **D** *Lobrathium lirunyui* **E** *Lobrathium pengi* **F** *Lobrathium uncinatum*.

## Supplementary Material

XML Treatment for Lobrathium ablectum

XML Treatment for Lobrathium anatinum

XML Treatment for Lobrathium bispinosum

XML Treatment for Lobrathium configens

XML Treatment for Lobrathium daxuense

XML Treatment for Lobrathium demptum

XML Treatment for Lobrathium diaoluoense

XML Treatment for Lobrathium dufui

XML Treatment for Lobrathium hebeatum

XML Treatment for Lobrathium hongkongense

XML Treatment for Lobrathium lirunyui

XML Treatment for Lobrathium pengi

XML Treatment for Lobrathium quyuani

XML Treatment for Lobrathium spathulatum

XML Treatment for Lobrathium taureum

XML Treatment for Lobrathium tortile

XML Treatment for Lobrathium tortuosum

XML Treatment for Lobrathium uncinatum
